# Cholecystokinin protects rats against *Staphylococcus aureus*-induced sepsis

**DOI:** 10.1186/cc11743

**Published:** 2012-11-14

**Authors:** RS Saia, FMC Zuelli, EC Cárnio

**Affiliations:** 1 University of Sao Paulo, Ribeirão Preto, São Paulo, Brazil

## Background

Cholecystokinin (CCK) was firstly described as a gastrointestinal hormone, but immune cells express their receptors, suggesting a possible involvement of this peptide in pathophysiological processes. Our aims were to evaluate the role of CCK on resistance against Gram-positive *Staphylococcus aureus*-induced sepsis, as well as cell influx to infectious focus. Furthermore, since nitric oxide, TNFα and IL-10 play a key role in the innate immune system controlling bacterial infection, we also evaluated the synthesis of these inflammatory mediators during this sepsis model.

## Methods

Male Wistar rats (180 to 200 g) received an intraperitoneal injection of proglumide (P) (nonselective CCK receptor antagonist; 30 or 50 mg/kg) 30 minutes before bacterial *S. aureus *inoculum (0.5 to 1 × 10^10 ^CFU/animal). At 4 and 24 hours after sepsis induction, blood and peritoneal lavage fluid (PLF) were collected for microbiological analysis, cytokines and nitrate quantifications and also differential cell counting. Nitrate was detected by chemiluminescence, while TNFα and IL-10 were determined by ELISA sandwich kits.

## Results

The pretreatment with P at higher dose (50 mg/kg) increased bacteremia in comparison with the saline-injected group (2,052 ± 810.7 vs. 154.3 ± 47.0 CFU/ml, *P *< 0.01) at 4 hours after sepsis induction. At the same time point, the bacterial counting in PLF increased in a dose-dependent manner in the P-treated rats (*P *< 0.05). On the other hand, only the higher P dose elevated significantly the CFU/ml in the PLF at 24 hours (97.75 ± 12.77 × 10^4 ^vs. 35 ± 10.05 × 10^4 ^CFU/ml, *P *< 0.05). The plasma TNFα and nitrate concentrations were not changed by treatment or time after sepsis induction. However, the administration of CCK receptors antagonist reduced the TNFα production in comparison with the control group in PLF, at both time points. The plasma IL-10 concentration increased at 4 hours in P-treated rats, while at 24 hours it was reduced (85.83 ± 48.0 vs. 1,698 ± 265.6 pg/ml, *P *< 0.001). In PLF, the rats pretreated with P reduced the IL-10 measurements (*P *< 0.05) when compared with the control group. In agreement, the macrophage influx to peritoneal infectious focus was compromised by treatment with a high P dose at 24 hours after *S. aureus*-induced sepsis (3,947.73 ± 269.99 vs. 5,629.61 ± 786.90 cells/μl; *P *< 0.05). Moreover, the neutrophil count did not change among the experimental groups (6,860.59 ± 211.90 vs. 6,273.54 ± 798.91 cells/μl).

## Conclusion

These data suggest a protective role for CCK peptide during *S. aureus*-induced sepsis, modulating the systemic and local inflammatory response, as well as increasing the macrophage influx to the infectious focus.

